# Public Perception of the Brain-Computer Interface Based on a Decade of Data on X: Mixed Methods Study

**DOI:** 10.2196/60859

**Published:** 2025-06-25

**Authors:** Mohammed A Almanna, Lior M Elkaim, Mohammed A Alvi, Jordan J Levett, Ben Li, Muhammad Mamdani, Mohammed Al‑Omran, Naif M Alotaibi

**Affiliations:** 1 College of Medicine, King Saud Bin Abdulaziz University for Health Sciences, Riyadh, Saudi Arabia; 2 King Abdullah International Medical Research Center, Riyadh, Saudi Arabia; 3 Department of Neurology and Neurosurgery, McGill University, Montreal, QC, Canada; 4 Division of Neurosurgery, Department of Surgery, University of Toronto, Toronto, ON, Canada; 5 Neuro International Collaboration, Toronto, ON, Canada; 6 Department of Neurologic Surgery, Mayo Clinic, Rochester, MN, United States; 7 Faculty of Medicine, University of Montreal, Montreal, QC, Canada; 8 Department of Surgery, University of Toronto, Toronto, ON, Canada; 9 Division of Vascular Surgery, St. Michael’s Hospital, Unity Health Toronto, University of Toronto, Toronto, ON, Canada; 10 Institute of Medical Science, University of Toronto, Toronto, ON, Canada; 11 Temerty Centre for Artificial Intelligence Research and Education in Medicine, University of Toronto, Toronto, ON, Canada; 12 Data Science & Advanced Analytics, Unity Health Toronto, University of Toronto, Toronto, ON, Canada; 13 Institute of Health Policy, Management and Evaluation, University of Toronto, Toronto, ON, Canada; 14 Institute for Clinical Evaluative Sciences, University of Toronto, Toronto, ON, Canada; 15 Li Ka Shing Knowledge Institute, St. Michael’s Hospital, Unity Health Toronto, Toronto, ON, Canada; 16 Leslie Dan Faculty of Pharmacy, University of Toronto, Toronto, ON, Canada; 17 College of Medicine, Alfaisal University, Riyadh, Saudi Arabia; 18 Department of Surgery, King Faisal Specialist Hospital and Research Center, Riyadh, Saudi Arabia; 19 National Neuroscience Institute, King Fahad Medical City, As Sulimaniyah, Makkah Road, Riyadh 12231, Riyadh, 59046, Saudi Arabia, 966 50 952 7700

**Keywords:** brain-computer interface, BCI, Neuralink, sentiment analysis, public perception, technology, innovation, Twitter, social media, natural language processing, NLP, mixed method, semantic, Mann-Kendall, brain-machine interface, decade, data, public perceptions, sentiment, marketing, education

## Abstract

**Background:**

Given the recent evolution and achievements in brain-computer interface (BCI) technologies, understanding public perception and sentiments toward such novel technologies is important for guiding their communication strategies in marketing and education.

**Objective:**

This study aims to explore the public perception of BCI technology by examining posts on X (formerly known as Twitter) using natural language processing (NLP) methods.

**Methods:**

A mixed methods study was conducted on BCI-related posts from January 2010 to December 2021. The dataset included 65,340 posts from 38,962 unique users. This dataset was subject to a detailed NLP analysis including VADER, TextBlob, and NRCLex libraries, focusing on quantifying the sentiment (positive, neutral, and negative), the degree of subjectivity, and the range of emotions expressed in the posts. The temporal dynamics of sentiments were examined using the Mann-Kendall trend test to identify significant trends or shifts in public interest over time, based on monthly incidence. We used the Sentiment.ai tool to infer users’ demographics by matching predefined attributes in users’ profile biographies to certain demographic groups. We used the BERTopic tool for semantic understanding of discussions related to BCI.

**Results:**

The analysis showed a significant rise in BCI discussions in 2017, coinciding with Elon Musk’s announcement of Neuralink. Sentiment analysis revealed that 59.38% (38,804/65,340) of posts were neutral, 32.75% (21,404/65,340) were positive, and 7.85% (5132/65,340) were negative. The average polarity score demonstrated a generally positive trend over the course of the study (Mann-Kendall Statistic=0.266; τ=0.266; *P*<.001). Most posts were objective (50,847/65,340, 77.81%), with a smaller proportion being subjective (14,393/65,340, 22.02%). Biographic analysis showed that the “broadcasting” group contributed the most to BCI discussions (17,803/58,030, 30.67%), while the “scientific“ group, contributing 27.58% (n=16,005), had the highest overall engagement metrics. The emotional analysis identified anticipation (score = 10,802/52,618, 20.52%), trust (score=9244/52,618, 17.56%), and fear (score=7344/52,618, 13.95%) as the most prominent emotions in BCI discussions. Key topics included Neuralink and Elon Musk, practical applications of BCIs, and the potential for gamification.

**Conclusions:**

This NLP-assisted study provides a decade-long analysis of public perception of BCI technology based on data from X. Overall, sentiments were neutral yet cautiously apprehensive, with anticipation, trust, and fear as the dominant emotions. The presence of fear underscores the need to address ethical concerns, particularly around data privacy, safety, and transparency. Transparent communication and ethical considerations are essential for building public trust and reducing apprehension. Influential figures and positive clinical outcomes, such as advancements in neuroprosthetics, could enhance favorable perceptions. The gamification of BCI, particularly in gaming and entertainment, also offers potential for wider public engagement and adoption. However, public perceptions on X may differ from other platforms, affecting the broader interpretation of results. Despite these limitations, the findings provide valuable insights for guiding future BCI developments, policy making, and communication strategies.

## Introduction

Brain-computer interface (BCI) is an emerging technology that allows for direct communication of the brain’s signals to external devices. This innovation operates through four sequential stages: signal acquisition, feature extraction, feature translation, and generating the device output [1]. The effectiveness of a BCI system largely depends on its signal acquisition module, which can range from noninvasive methods, such as surface electroencephalography electrodes, to more invasive approaches like endovascular stent-electrode arrays and electrodes implanted on the brain surface. While more invasive methods generally allow for higher signal fidelity and better system performance, overall effectiveness also depends on additional factors such as signal processing and user adaptability [2,3].

BCI has been extensively researched across various fields, including medical rehabilitation [4-6], control of orthotic and prosthetic devices [7,8], assistive technologies [9,10], and video gaming [11]. Additionally, BCI has potential applications in enhancing cognitive functions [12]. The remarkable progress in BCI, along with the involvement of well-recognized institutions and prominent figures like Elon Musk, has brought this technology to the forefront of public awareness. Public perception of BCI technologies is influenced by their representation in the media, with concerns over ethical dilemmas including privacy and mind control, and the invasive nature of certain BCI technologies [13-18].

X (formerly known as Twitter) provides real-time insights into the thoughts, feelings, and conversations of millions of users. Natural language processing (NLP) tools are instrumental in analyzing social media content, offering deeper insights into public perception. NLP methods enable the analysis of public sentiment toward specific topics, the detection of emerging trends, and the identification of demographic groups participating in these discussions. These tools have been extensively used to assess public acceptance of vaccines [19,20], guide economic investments [21], evaluate innovative products [22,23], and more. BCI is an emerging technology with concepts that many may still consider science fiction, leading to polarized opinions among the public. While some individuals might be excited about its potential applications, others may express concerns due to potential complications and the possibility of malicious uses. Understanding public sentiment toward BCI is crucial for guiding ethical frameworks, informing policy decisions, and shaping the direction of future research and development. However, there is a lack of comprehensive studies assessing public perception of BCI through social media discussions. This study aims to bridge the gap by using NLP tools to analyze over a decade of X conversations about BCI. The goals of this study are to quantify sentiments, identify trends in public perception, explore subjectivity, and understand the nature of public discussions related to BCI.

## Methods

### Data Source and Processing

We used X application programming interface, Twitter application programming interface for academic research, and database to identify posts related to BCI using the search term “brain-computer interface.” The acronym “BCI” was not used to avoid including irrelevant posts. The search was conducted from X inception (March 2006) to May 2022, prior to Elon Musk’s acquisition of X. The data underwent preprocessing, which involved omitting any mentions, URLs, and hashtags, removing any line breaks, deleting any HTML characters, replacing them with their respective Unicode equivalent, eliminating any special characters or punctuation points except exclamation points (the only punctuation mark relevant for sentiment analysis), and excluding posts from users with fewer than 10 followers to minimize “bot” influence and duplicate entries. We excluded posts before January 2010 due to limited data availability and after December 2021 to maintain the temporal consistency of the dataset, as our data cover only a few months of 2022 (Figure S1 in Multimedia Appendix 1). Detailed individual post data included the text, date and time of posts, the number of reposts, replies, likes, and quote count. Additional data included whether the post included links, media, tagging, or any hashtags. User information included username, the number of followers, the total number of author posts, user biography, and location.

### Biography Analysis

To explore users’ demographics, we used the sentiment.ai [24] library match function after preprocessing user biographies and excluding users with empty biographies. Sentiment.ai is a text-based deep machine learning tool that allows for category matching of the most similar phrase and its category, providing a cosine similarity score. The categories (ie, the biographic groups) and phrases (ie, attributes) are shown in Table S1 in Multimedia Appendix 1. We applied categories such as “broadcast,” “scientific,” “entrepreneurship,” and “clinical” based on predefined attributes. We measured the cosine similarity score between profile biographies and the most similar attribute, categorizing them as “others” if the similarity score is less than 0.05 [23] (Figure S2 in Multimedia Appendix 1).

### Sentiment Quantification

#### Sentiment by Valence Aware Dictionary and Sentiment Reasoner

The sentiment polarity was analyzed using the VADER (Valence Aware Dictionary and Sentiment Reasoner) library [25], a lexicon designed for sentiment analysis in social media contexts. It accounts for important elements including emojis, emoticons, slang words, and acronyms or initialisms with sentimental value (eg, “lol”) in determining the compound score. The compound score is calculated by adding the sentiment scores of each word, and it is set to be between −1 (negative) and +1 (positive) after being adjusted according to a set of rules. The threshold in our study was a compound score of ≥0.05 for positive sentiment posts, <0.05 and >−0.05 for neutral sentiment posts, and ≤−0.05 for posts expressing negative sentiment (Figure S3 in Multimedia Appendix 1).

#### Emotions by NRCLex

The NRCLex tool [26] is a Python library that allows for the analysis of the emotional content of text using the National Research Council Canada emotion lexicon, which contains approximately 27,000 words. It provides a simple interface to extract various emotions and sentiments from text. The emotional effects measured include fear, anger, anticipation, trust, surprise, sadness, disgust, and joy. In our study, we used the “raw_emotion_scores” model of the NRCLex tool to count the frequency of words associated with certain emotions in a text. For example, a post containing 3 words associated with anticipation and 1 word associated with trust would have an emotion score of +3 for anticipation and +1 for trust. The primary emotion for each post was determined by identifying the emotion with the highest score, giving equal consideration to multiple emotions if they had equal scores. If no emotion was identified for a post, we used the label “no emotion” and excluded such posts from any emotion analysis. These analyses were applied only to posts with positive or negative sentiment, excluding posts with neutral sentiment, as they are not informative for emotion analysis (Figure S3 in Multimedia Appendix 1).

#### Subjectivity by TextBlob

TextBlob library [27] was used to classify X posts based on the subjectivity score (ranging from 0 to 1), which detects the degree of personal opinion expressed in the text. Words that are more opinion-based (eg, scary and amazing) have a higher subjectivity score, whereas words that are fact-based (eg, data and communication) have a lower subjectivity score. The threshold used in our study is a subjectivity score of 0.5. If the score is greater than or equal to 0.5, the post is labeled as “subjective,” otherwise the post is labeled as “objective.” For nontemporal analyses, we performed sensitivity analyses to verify the results by excluding duplicated texts (ie, similar text posted by different users) and conducted the sentiment and emotion analyses on 1000 randomly selected posts (Figures S3 and S4 in Multimedia Appendix 1).

### Trends in Public Perception

Changes in sentiments were tracked over time. The Mann-Kendall trend test was applied to identify significant trends in monthly incidence. Temporally weighted analyses were used to identify any changes in users’ discussions and comments regarding BCI technologies over time.

### Topic Modeling

To better understand the context of topics within the complex discussion regarding BCI in X, we used the BERTopic tool [28] to conduct a topic modeling analysis. BERTopic is a topic modeling technique that uses deep learning and NLP to better understand the context and semantic relationships within text data generating clusters of similar texts, interpreted as topics. It works by embedding text into numerical representations, which are converted into a high-dimensional vector using a pretrained transformer model. Text or sentences related to each other will be close to each other in this vector, while unrelated ones will be away from each other. After dimensionality reduction of the vector, the lower-dimensional embeddings are clustered to group similar documents together. Each cluster represents a potential topic. Finally, each topic is represented by its most representative documents or by extracting keywords that best describe the cluster. Results from this topic modeling analysis include the frequency of certain words within a topic and the probability that given words represent certain topics. We preprocessed the data by removing duplicate posts, then used the “all-MiniLM-L6-v2” embedding model and the SentenceTransformer model to analyze topics in the posts discussing BCI. The top 8 topics discussed in our dataset are visualized in topic word score bar charts, including specific topics for posts expressing positive or negative sentiments alone (Figure S4 in Multimedia Appendix 1)

### Statistical Analysis

All analyses were conducted using Python (version 3.8; Python Software Foundation) and R (version 4.4.0; R Foundation for Statistical Computing). The *P* value threshold for statistical significance in this study is set at .05.

### Ethical Considerations

All extracted data used and presented in this study were archival, cross-sectional, and observational, obtained from publicly accessible sources without any interaction with social media users and with their usernames omitted. As such, institutional review board approval was not required.

## Results

### Post Characteristics

Our study analyzed a total of 65,340 posts, created by 38,962 distinct users (Table 1). These users had a median follower count of 662 (IQR 173-2332). The median number of total posts per user was 8976.5 (IQR 2294-33,677). Most of the posts came from a diverse user base, as only 5.21% (3405/65,340) originated from the top 50 most active contributors. The content of these posts varied, as 60,079 (91.94%) included links, while 5838 (8.93%) featured media. A total of 16,617 (25.43%) posts contained tags, and 18,623 (28.5%) posts contained hashtags. Engagement metrics showed that 17,141 (26.23%) posts received at least 1 like, 5104 (7.81%) posts had at least 1 reply, 12,210 (18.68%) posts were reposted at least once, and 2688 (4.11%) posts contained quote posts. Post characteristics from 1000 randomly selected posts and a text duplicate–free sample are showcased in Table 1.

**Table 1.** Overview of post characteristics with complete dataset and validation subsets in BCI discussions on X.

Post characteristics	Total posts (n=65,340)^a^,n (%)	Duplicate texts removed (n=39,990)^b^,n (%)	Random 1000 posts^c^,n (%)
At least 1 repost	12,210 (18.68)	8710 (21.78)	170 (17)
At least 1 reply	5104 (7.81)	4203 (10.51)	78 (7.8)
At least 1 like	17,141 (26.23)	12,640 (31.60)	255 (25.5)
At least 1 quote	2688 (4.11)	1849 (4.62)	32 (3.2)
Contains a link	60,079 (91.94)	35,020 (87.57)	913 (91.3)
Contains a media	5838 (8.93)	4218 (10.45)	80 (8)
Contains a tagging	16,617 (25.43)	12,122 (30.31)	254 (25.4)
Contains a hashtag	18,623 (28.50)	13,336 (33.34)	281 (28.1)

a The number of total unique users: 38,962.

b The number of unique users of the duplicate-free subset: 25,008.

c The number of unique users of the random 1000 posts subset: 959.

### User Biography

Biographic analysis (unique users=34,565) showed that “broadcasting” (10,171/34,565, 29.42%) was the highest group, followed by “entrepreneurship” (9359/34,565, 27.02%), “scientific” (9200/34,565, 26.61%), “clinical” (4066/34,565, 11.76%), and “other” (1769/34,565, 5.11%). The contribution to the BCI discussion (total posts=58,030) was the highest from users in the “broadcasting” group with 17,803 (30.67%) posts, followed by “scientific” with 16,005 (27.58%) posts, “entrepreneurship” with 14,008 (24.13%) posts, “clinical” with 7380 (12.71%) posts, and “other” with 2834 (4.88%) posts (Table 2). The “scientific” group had the highest engagement metrics among all the biography groups, while the “entrepreneurship” group had the lowest engagement metrics in our dataset (Table 3).

**Table 2.** Distribution of unique users and total posts by biographic group in brain-computer interface discussions on X.

Biographic group	Unique users^a^ (n=34,565), n (%)	Total posts^a^ (n=58,030), n (%)
Broadcasting	10,171 (29.42)	17,803 (30.67)
Entrepreneurship	9359 (27.02)	14,008 (24.13)
Scientific	9200 (26.61)	16,005 (27.58)
Clinical	4066 (11.76)	7380 (12.71)
Other	1769 (5.11)	2834 (4.88)

a Users with empty user biography were excluded from the analysis.

**Table 3.** Post characteristics and engagement metrics across biographic groups in brain-computer interface discussions on X.

Post characteristics	Broadcasting^a^, n (%)	Entrepreneurship^b^, n (%)	Scientific^c^, n (%)	Clinical^d^, n (%)
At least 1 repost	3549 (19.93)	2456 (17.53)	3675 (22.96)	1601 (21.69)
At least 1 reply	1596 (8.96)	986 (7.03)	1511 (9.44)	545 (7.38)
At least 1 like	4822 (27.08)	3571 (25.49)	5119 (31.98)	2266 (30.70)
At least 1 quote	934 (5.24)	482 (3.44)	831 (5.19)	286 (3.87)
Contains a link	16,307 (91.59)	13,058 (93.21)	14,568 (91.02)	6913 (93.67)
Contains a media	1628 (9.14)	1264 (9.02)	1600 (9.99)	730 (9.89)
Contains a tagging	4128 (23.18)	3757 (26.82)	4766 (29.77)	1834 (24.85)
Contains a hashtag	4990 (28.02)	4655 (33.23)	4687 (29.28)	2289 (31.01)

a 17,803 (30.67%) posts.

b 14,008 (24.13%) posts.

c 16,005 (27.58%) posts.

d 7380 (12.71%) posts.

### Engagement Trends

**Figure 1.**
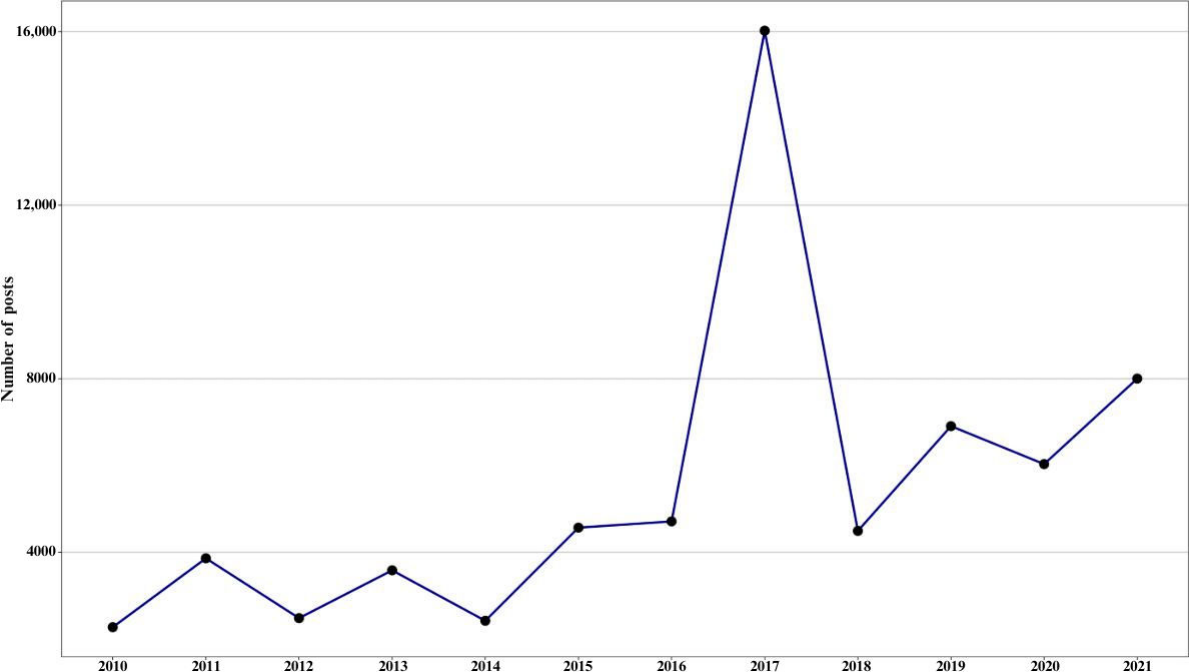
The number of posts shared on X discussing brain-computer interface (BCI) annually from 2010 to 2021. From 2010 to 2014, the number of posts remained relatively steady, fluctuating but less than 4000 per year. A gradual increase began in 2015, peaking sharply in 2017 at around 16,000 posts, marking the highest level of activity in the timeline. This spike coincides with the public announcements of Elon Musk’s BCI company, Neuralink, and Facebook’s BCI project. Following this peak, there was a substantial drop in 2018, with post numbers returning to earlier levels. From 2019 to 2021, the number of posts showed a fluctuating yet gradual increase.

**Figure 2.**
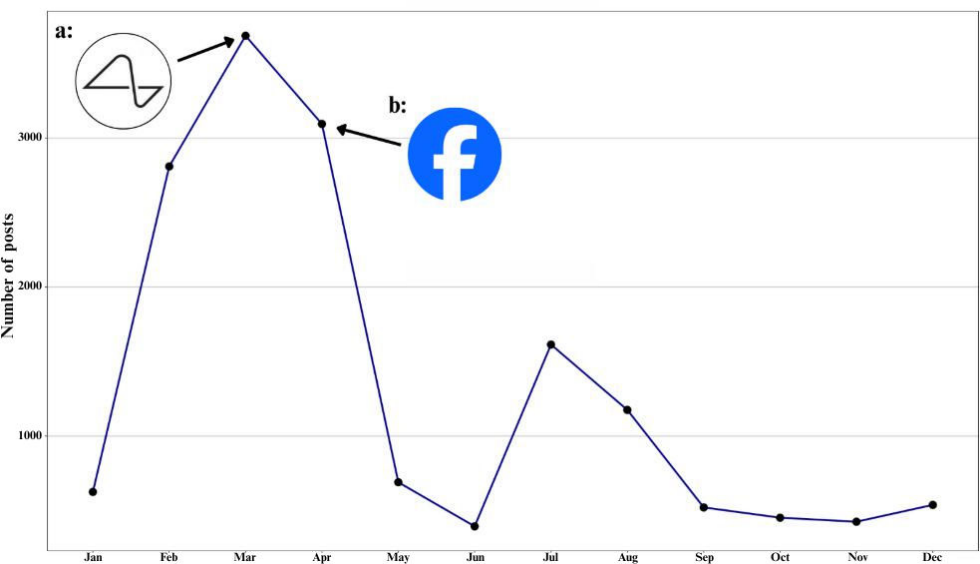
The number of posts shared on X discussing brain-computer interface (BCI) per month of the year 2017. The number of posts increases from January, peaking in March and April at over 3000 posts each month. The peak in March (a) coincides with Neuralink’s public announcement, while the peak in April (b) aligns with Facebook’s announcement of its BCI project. Following these peaks, there is a sharp decline in May, with the number of posts dropping to the lowest point in June. A slight increase is observed in July, but the number of posts remains lower than the earlier peaks, stabilizing at lower levels from September to December.

In 2017, there was a substantial increase in the number of posts, which accounted for approximately 24.52% of the entire dataset (Figure 1). March had the highest number of posts (n=3686), followed by April (n=3094) (Figure 2). This rise in March’s posts was primarily due to Elon Musk’s announcement of “Neuralink,” his BCI company [29]. The words “Neuralink,” “Musk,” and “Elon” were collectively mentioned 5831 times in March. The number of posts in April can be attributed to the announcement of Facebook’s BCI projects, with the term “Facebook” receiving 1944 mentions [30]. A bar chart containing the 25 most frequently mentioned terms, excluding BCI-related terms is included in Figure S5 in Multimedia Appendix 1.

### Sentiment Quantification and Trends in Public Perception

**Figure 3.**
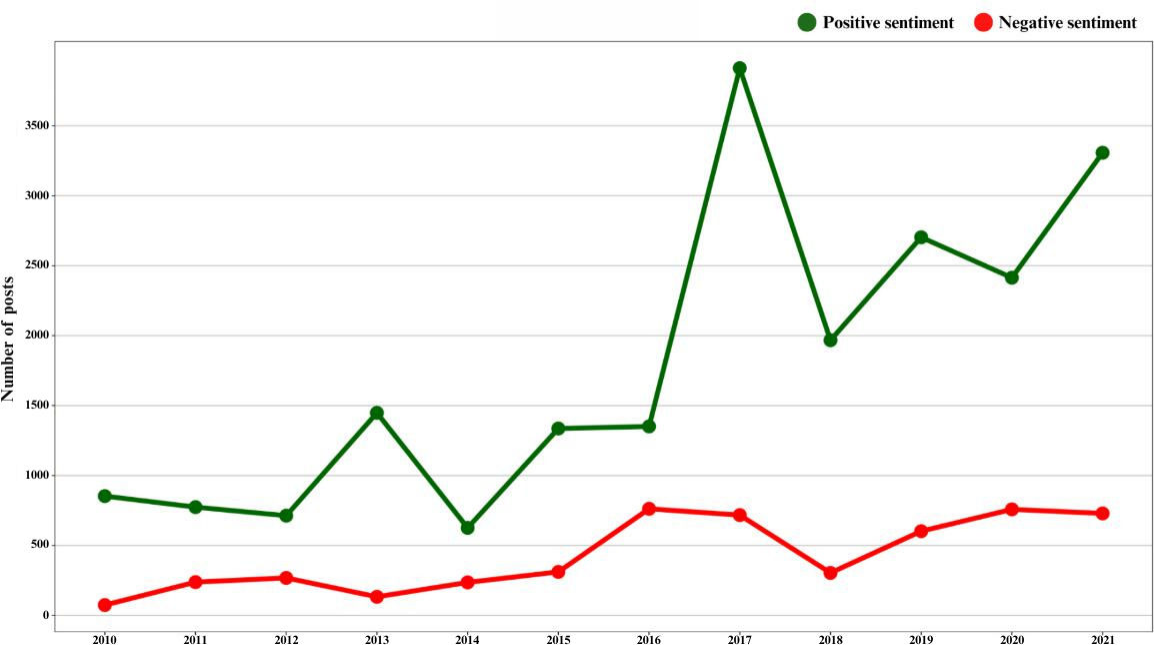
The annual number of brain-computer interface–related posts on X from 2010 to 2021, categorized by sentiment. Positive sentiment posts (green line) increased significantly from 2016, peaking in 2017 at over 3500 posts, followed by a decline in 2018 and subsequent growth through 2021. Negative sentiment posts (red line) remained relatively steady throughout the period, showing minor fluctuations but consistently lower numbers compared to positive sentiment posts. Overall, positive sentiment posts were more prevalent than negative ones across all years.

Most of the posts tended to express neutral sentiments (38,804/65,340, 59.38%). Approximately one-third of the posts conveyed positive sentiments (21,404/65,340, 32.75%), while a smaller portion expressed negative sentiments (5132/65,340, 7.85%). Deletion of duplicate text resulted in a notable polarization of sentiments, potentially due to the exclusion of posts containing only titles of news articles, which tend to be more neutral in tone (Table 4). A considerable increase in positive posts was observed in February 2017, coinciding with the publication of Stanford-led BCI studies, and in July 2017, following the Defense Advanced Research Projects Agency (DARPA) award for BCI [31-33]. The number of posts expressing negative sentiment remained low, with an average of 428 (8.19%) posts per year throughout the study period (Figure 3; Figure S6 in Multimedia Appendix 1). The average polarity score showed an overall positive trend throughout the study (Mann-Kendall Statistic=0.266; τ=0.266; *P*<.001; Figure S7 in Multimedia Appendix 1). The mean sentiment score increased substantially upon the announcement of the collaboration between Stanford University and BrainGate in November 2011, in April 2016 following the publication of the study on the use of BCI in restoring functional movement in apatient with quadriplegia, and again in July 2017 with the DARPA-led award [33-36]. The spike in negative sentiment posts in March 2017 was mostly due to discussion related to Musk’s involvement in BCI, with an excited yet conservative tone. The word “help” (n=1489 mentions) was common in posts expressing positive sentiment, whereas in posts with negative sentiment, the term “injury” (n=325 mentions) was the most mentioned word. The frequent mentions of Elon Musk and artificial intelligence (AI) in the positive and negative sentiments are suggestive of mixed and polarized opinions about him and his BCI company “Neuralink” (Figure S8 in Multimedia Appendix 1). Most of the posts were objective (50,847/65,340, 77.81%), while fewer were subjective (14,393/65,340, 22.02%; Table 4).

**Figure 4.**
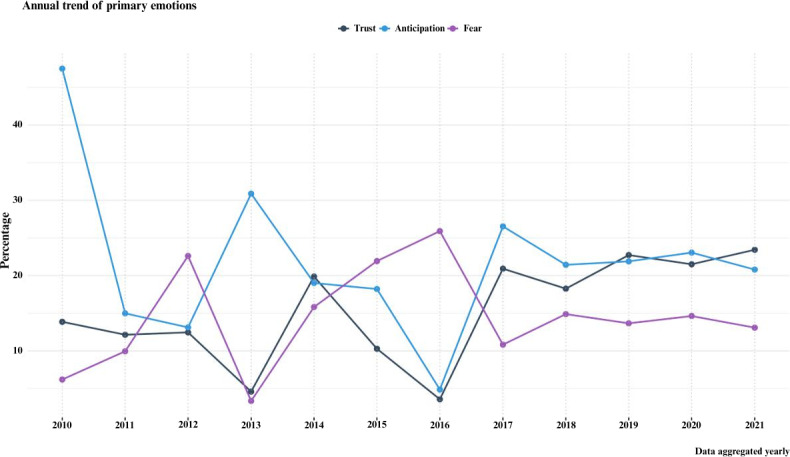
The annual trend of primary emotions—trust, anticipation, and fear—expressed in posts discussing brain-computer interface on X from 2010 to 2021. It indicates fluctuations in the presence of each emotion over time. Anticipation (light blue line) shows varied peaks and troughs throughout the timeline, with prominent peaks in 2010, 2013, and 2017. Trust (dark blue line) displays a more consistent pattern with smaller fluctuations and an increase toward the end of the period. Fear (purple line) remains relatively stable over the years, with slight peaks in 2012, 2015, and 2016, but generally shows lower percentages compared to anticipation and trust.

The most prevalent emotion scores observed were “anticipation,” “trust,” and “fear,” accounting for 20.52% (10,802/52,618), 17.56% (9244/52,618), and 13.95% (7344/52,618) of the expressions, respectively. However, “surprise,” “joy,” and “disgust” were less frequently expressed, constituting 8.77% (4619/52,618), 12.69% (6681/52,618), and 2.79% (1470/52,618), respectively (Table 5). The emotion score of the text duplicate–free dataset and random 1000 posts are in Table 5. Focusing on the 3 most prominent emotions, “anticipation” peaked at 47.49% in 2010, coinciding with the sharing of news regarding the potential gamification of BCI technologies [37]. In July 2017, there was a substantial increase in posts expressing anticipation as their primary emotion reaching as high as 48.07% of posts shared, discussions were mostly related to DARPA’s award for BCI, and the use of BCI in the production of music using the “encephalophone” [33,38,39]. “Trust” reached 23.43% of total posts in 2021 and was the lowest in 2016 with 3.57% of total posts. “Fear” displayed a notable increase in posts in 2016, with 25.90%, and was the lowest in 2013 with 3.34% (Figure 4; Figure S9 in Multimedia Appendix 1).

**Table 4.** Overview of sentiment and subjectivity in brain-computer interface discussions on X across the complete dataset and validation subsets.

	Total posts (n=65,340), n (%)	Duplicate texts removed (n=39,990), n (%)	Random 1000 posts, n (%)
Sentiment			
Positive sentiment	21,404 (32.75)	15,390 (38.48)	334 (33.4)
Neutral sentiment	38,804 (59.38)	20,924 (52.32)	591 (59.1)
Negative sentiment	5132 (7.85)	3676 (9.1)	75 (7.5)
Subjectivity			
Subjective	14,393 (22.02)	10,571 (26.4)	230 (23)
Objective	50,847 (77.81)	29,419 (73.5)	770 (77)

**Table 5.** Distribution of positive and negative emotions in brain-computer interface discussions on X across the complete dataset and validation subsets.

Emotion	Total posts, score^a^^,^^b^ (%)	Duplicated text removed^c^^,^^b^, score (%)	Random 1000 posts,^b^^,^^d^ score (%)
**Positive emotions**			
Anticipation	10,802 (20.52)	7855 (20.52)	454 (22.07)
Trust	9244 (17.56)	7536 (19.69)	386 (18.76)
Joy	6681 (12.69)	5150 (13.45)	265 (12.88)
Surprise	4619 (8.77)	3261 (8.52)	174 (8.45)
**Negative emotions**			
Fear	7344 (13.95)	5083 (13.28)	274 (13.32)
Sadness	6623 (12.58)	4402 (11.50)	244 (11.86)
Anger	5745 (10.91)	3646 (9.52)	185 (8.99)
Disgust	1470 (2.79)	1317 (3.44)	74 (3.59)

a Total score, excluding posts expressing no emotion and neutral sentiment: 52,618

b Emotions conveyed equally with similar scores are considered separately.

c Total score, excluding posts expressing no emotion and neutral sentiment: 38,268

d Total score, excluding posts expressing no emotion and neutral sentiment: 2057

### Temporally Weighted Sentiment Analyses

A temporally weighted analysis was conducted to explore the change in public discussion before, during, and after the spike in 2017 (Table 6). The increase in posts expressing a neutral sentiment (11,394/16,023, 71.11%), as well as the rise in posts expressing anticipation (1417/2512, 56.41%) and trust (n=1117/2512, 44.47%) in 2017, demonstrates a public that is excited yet cautious toward this emerging technology. However, after 2017, the public discussion polarized, as positive and negative views increased substantially, from 24.41% (3912/16,023) to 40.86% (10,391/25,430) and from 4.47% (717/16,023) to 9.41% (2393/25,430), respectively. Additionally, there was a notable increase in subjective posts, from 18.95% (3036/16,023) to 26.76% (6804/25,430), after 2017. Fear was substantially expressed before 2017 with 40.97% (1936/4725), then dropping to below 30% during and after 2017, possibly indicating the public excitement, and trust following the big announcements in the BCI industry in 2017. However, the increase from 23.01% (578/2512) to 28.3% (2237/7904) in fear might indicate unaddressed ethical questions.

**Table 6.** Sentiment, subjectivity, and primary emotions over time in brain-computer interface discussions on X.

	Before 2017 (n=23,887), n (%)	During 2017 (n=16,023), n (%)	After 2017 (n=25,430), n (%)
**Sentiment**			
Positive	7101 (29.73)	3912 (24.41)	10,391 (40.86)
Neutral	14,764 (61.81)	11,394 (71.11)	12,646 (49.73)
Negative	2022 (8.46)	717 (4.47)	2393 (9.41)
**Subjectivity**			
Objective	19,234 (80.52)	12,987 (81.05)	18,626 (73.24)
Subjective	4653 (19.48)	3036 (18.95)	6804 (26.76)
**Emotions** ^a^ ^,b^			
Anticipation	1977 (41.84)	1417 (56.41)	3490 (44.15)
Trust	913 (19.32)	1117 (44.47)	3506 (44.36)
Fear	1936 (40.97)	578 (23.01)	2237 (28.30)

a Posts expressing no primary emotion were excluded from the analysis. Emotions conveyed equally with similar scores are considered separately; hence, percentages exceeding 100%.

b Before 2017: 4725 posts, during 2017: 2512 posts, and after 2017: 7904 posts.

### Topic Modeling

We conducted a topic modeling analysis to understand the semantic and dynamic discussion related to BCI technology. The main topics discussed in our dataset included ideas related to BCI changing the future, Musk’s announcement of “Neuralink,” Facebook’s involvement in BCI technology, and the gamification of BCI technologies (Figure S10 in Multimedia Appendix 1). Topics discussed in posts expressing positive sentiment included the advancement of BCI in research and health care and BCI advancements with virtual reality (VR) and augmented reality (AR; Figure S11 in Multimedia Appendix 1). Discussions in posts conveying negative sentiment were related to conspiracy theories of Musk or “Neuralink,” further demonstrating that the involvement of Musk in BCI was although highly influential but also particularly controversial. Moreover, our analysis detected discussions related to the potential use of BCI for malicious reasons including mind control (Figure S12 in Multimedia Appendix 1). Examples of posts related to different topics are included in Table S2 in Multimedia Appendix 1.

## Discussion

### Principal Findings

Our study highlights the complex and evolving public perception of BCI technology, as expressed on X discussions over the past decade. Sentiment analysis reveals that while a majority of the public holds neutral views, there is a notable undercurrent of anticipation, trust, and fear. The presence of these conflicting emotions suggests that while there is optimism about the potential of BCIs, concerns around ethical issues remain unresolved. Understanding these emotions is essential for the development and acceptance of BCI technology. Key topics that emerged from the discussions include Neuralink and Elon Musk—subjects of both positive and negative conversations—the practical applications of BCIs, and the potential for gamification of BCI technology. In addition to being key market drivers, addressing these factors will influence innovation and drive investments in the BCI space.

### Ethical Challenges

BCI is one of the most important technologies in neuroscience, sparking diverse opinions regarding its ethical implications [40-42]. Safety, justice, privacy, and security are the main concerns discussed in ethical literature [43]. Some authors had concerns over the safety of implanting such invasive technologies, which could potentially cause serious health complications [44], while others have criticized the biased narrative in the BCI literature, which tends to overlook the perspectives of individuals with disabilities [45]. There was unease about the handling and storage of sensitive data obtained through BCI, with worries about its potential exploitation for malicious purposes [46]. In our study, we detected discussions reflecting many of these concerns. We specifically highlighted concerns regarding Elon Musk and his company Neuralink, particularly related to safety and animal rights issues [47], as well as the potential for BCI to be used in terrorism, or for brain hacking, which could lead to the leaking of sensitive personal information or even revealing emotions and thoughts. Open and inclusive discussions are essential to guiding the ethical development and use of BCI technology. Moreover, users from the scientific community will play a particularly critical role in this process. Our study found that posts from scientists generated the highest engagement when discussing BCI, underscoring their influence in shaping public understanding. By leveraging their trusted position, scientists can educate and foster informed discourse on these ethical issues to steer the responsible development and application of BCI technology.

### Elon Musk’s Involvement

The significant rise in digital discussions about BCI in X, particularly in March 2017, aligns with Elon Musk’s announcement of his BCI company, Neuralink. Elon Musk’s substantial social media influence is evident, with almost 200 million followers on X*.* His posts not only are influential but often polarize public opinion [48]. For instance, his comments on cryptocurrencies have demonstrably impacted their market values [49]. Additionally, a sentiment analysis focused on electric vehicles revealed that Musk was a central figure in these discussions [50]. These observations reinforce the significant role of influential figures like Elon Musk in steering the public dialogue and development of cutting-edge technologies.

Most posts conveyed a neutral sentiment, reflecting a combination of anticipation and excitement, yet mixed with fear and anxiety, resulting in a generally doubtful public perception. This perception could potentially be the result of unanswered ethical challenges. Furthermore, Musk’s involvement in BCI technologies has significantly influenced public perceptions, often polarizing opinions. The 2023 sentiment analysis conducted on Reddit indicates that neutral and negative views toward Elon Musk outnumber positive sentiments in discussions about him [48]. Additionally, in our study, a common theme in both the negative and positive discussions about BCI frequently centered around Musk or Neuralink. This mixed perception of Elon Musk could be another contributing factor to the sentiment results of our study. Ensuring transparent communication from influential figures like Musk might be key to positively shifting public sentiment toward BCI technologies.

Since acquiring Twitter, now rebranded as X, Elon Musk has become actively involved in geopolitical discourse, leading to conflicting opinions about his involvement in politics. This increased political engagement has further polarized public perception of him [51,52], which might potentially extend to BCI technologies. To mitigate this migration of polarization, it is essential to diversify the voices in BCI communication by encouraging scientists and industry experts to take a more prominent role, thereby reducing the focus on any single individual.

Nonetheless, Musk’s involvement in BCI technology has had a significant and positive impact, contributing to greater public awareness and advancements in the field. The surge in BCI-related discussions in 2017, following Musk’s announcement of Neuralink, not only increased awareness of the technology and its potential applications but also led to a rise in posts expressing positive sentiments, mixed with anticipation and trust. This suggests a general optimism regarding the potential of BCI technology. Furthermore, this trust and enthusiasm is reflected in the recent growth of BCI-related publications as well as the rapid expansion of the BCI market [53,54]. In summary, even though Musk is considered a controversial figure by many, his influence in the field of BCI has been largely positive. However, it is important to continue making conscious efforts to ensure that this positive impact is sustained and that the technology develops responsibly and ethically.

### Bridging Public Sentiment and Practical Applications

We found that anticipation and trust were the most expressed emotions, likely stemming from the involvement of renowned universities in BCI research, including the collaborative BrainGate2 project [34,35]. The widespread media coverage and remarkable BCI outcomes in individuals with severe disabilities have also contributed to this trend [31,32,36]. These technologies enable patients with disability to overcome their disabilities, thereby enhancing their quality of life. The public enthusiasm observed toward these milestones underscores a substantial societal demand for such innovations. This heightened positive sentiment indicates that the integration of BCIs into neuroprosthetics, AI-driven prosthetics, and exoskeleton devices not only represents a significant technological advancement but also fulfills a critical public need for effective solutions to address disabilities. Moreover, BCI technology used for nonrehabilitation activities, such as gaming and music production, led to an increase in anticipation due to its potential appeal and application to everyday consumers.

Fear was the third most prevalent emotion identified. This may be related to ongoing unanswered ethical concerns in BCI development, varying public opinions about public figures, and inherent fear toward novel technologies. Our capture of fear might be an early indicator of potential technophobia, as BCI becomes more commercially available. Difficult to understand and complex technologies are associated with more anxiety and fear of using them [55]. In addition, the concept of “mind control,” frequently portrayed in popular culture and science fiction, could potentially heighten fear perceptions. Upcoming depictions and future works that continue to explore this theme may trigger more apprehensive emotions. Such emotion, if not addressed, could foster a conservative mindset, potentially slowing the adoption and hindering the application of innovative technologies such as BCI.

### The Gamification of BCI Technologies

One particularly exciting avenue for BCI technology is its integration into gaming. The potential of gamification in BCI technologies is vast, offering applications not only for medical rehabilitation [56,57] but also for entertainment experiences [58-60]. The concept of controlling and fully immersing oneself in a game to create experiences beyond physical limitations is highly appealing to the public, as evidenced by a notable increase in anticipation. Gabe Newell, cofounder and president of Valve, showcased Valve’s interest in BCI for gaming, sparking significant enthusiasm [61]. Integrating BCI technologies into gaming could play an important role in further advancing their development, much like the early adoption of VR and AR through gaming platforms such as the HTC Vive (by HTC and Valve Corporation), Meta Quest (by Meta Platforms), and PlayStation VR (by Sony Interactive Entertainment). These technologies, which began in gaming, have since expanded into broader applications in healthcare and education.[62]. Fueled by public enthusiasm, several companies have begun claiming that their products leverage BCI to enhance gaming experiences. However, it remains debatable whether we have truly reached that point [11]. Numerous attempts have been made to use BCI for gaming, using both invasive and minimally invasive technologies. For example, Neuralink has successfully implemented a BCI in a human with quadriplegia, allowing the individual to play chess and other games by moving a cursor [63]. “I basically have an aimbot in my head,” said Noland Arbaugh, the first patient to receive a brain-computer chip from Neuralink. An “aimbot” is a type of cheating software used in video games that automatically aims at opponents at a superhuman speed [64,65]. Similarly, a popular Twitch streamer successfully beat the notoriously difficult game *Elden Ring* using a wearable electroencephalograpy-based BCI device, with thousands of live viewers [66]. With a global video game market size estimated at US $217.06 billion in 2022, these advancements are likely to positively influence public perception of BCI and may attract the attention of major players in the gaming industry, potentially driving further research and development of BCI technologies [67]. Gamification thus presents a significant opportunity for the future of BCIs, acting as a gateway for mainstream consumer adoption, similar to how VR or AR technologies gained traction.

### Public Acceptance and Trust: Key Drivers for the Growing BCI Market

The economic outlook for BCIs is promising. In 2023, the global market for BCIs was valued at US $2.0 billion and is projected to grow at a compound annual growth rate of 17.8% from 2024 to 2030. This growth is driven by increasing demand for neuroprosthetic devices and advancements in technology that enhance mobility and communication for patients who are paralyzed as well as expanding applications in gaming and military communication [53]. However, public opinion and acceptance are essential to sustaining this market growth. Greater acceptance of the technology will drive further adoption and development, especially since young investors and future philanthropists are likely to support companies that align with their personal values and contribute to humanitarian goals [68]. Our findings align with this broader market analysis. Most individuals in our study exhibit a neutral sentiment toward BCI technology, reflecting a phase of critical appraisal of this emerging innovation. Additionally, the prominence of emotions such as trust, anticipation, and fear underscores the imperative to educate, demystify, and familiarize the public with BCI technology. Doing so will be key to enhancing acceptance among the public. Additionally, the increase in the number of subjective posts in our analysis reflects increased awareness of BCI as a topic for discussion, and this growing awareness suggests that the public will begin to form their own ideas, opinions, and emotions toward BCI. This awareness might also indicate that people are shifting from passive observers to active participants in BCI discussions, contributing to the evolving conversation and potentially influencing its trajectory. Therefore, a concerted effort to inform and engage the public is crucial for fostering positive sentiment and ensuring the responsible development and integration of BCI technology. This, in turn, will support the continued expansion of the global BCI market.

### Limitations

The study has several limitations that may have impacted the accuracy of our results. First, the search excluded the term “BCI” to avoid including irrelevant posts, which may have inadvertently omitted relevant content. Additionally, the analysis was restricted to English-language posts and focused solely on the platform X, potentially overlooking important discussions on other platforms or in different languages. It was not possible to infer some of the important demographics of users missing out on important contextual information. We used existing tools for NLP analyses that were not tailored and validated specifically for the study’s topic, which may potentially lead to inaccurate results. Determining the geographical locations of users was not feasible, which would have provided valuable regional insights. Regarding the biography analysis, some overlap between categories is expected, as a single user may fall into multiple classifications. This method of short bioclassification is effective for only a subset of users, and further validation is required in future studies. Finally, the substantial number of posts containing links may indicate that the sample is not representative of general discussions but primarily consists of shared news articles. Future research should address these limitations to enhance the depth and accuracy of the understanding of the public perception toward BCI technologies.

### Conclusions

The findings from this NLP-assisted study offer a decade-long overview of public perception of BCI technology. Overall sentiment was mostly neutral, but the emotions most commonly linked to BCI—anticipation, trust, and fear—reflect a complex emotional response, suggesting a cautiously optimistic yet apprehensive attitude toward the advancement of BCI technology. Notably, the presence of fear underscores the importance of addressing ethical concerns and ensuring transparent communication within the BCI field. Resolving these issues is critical for reassuring the public and mitigating apprehensive attitudes. Additionally, the involvement of influential figures and leading institutions, along with reports of positive clinical outcomes, such as advancements in neuroprosthetics and rehabilitation, may foster more favorable public perceptions of BCI technology. The gamification of BCI, particularly its integration into gaming and entertainment, also offers a pathway to increased public engagement and adoption. However, the persistence of fear signals potential resistance that may impede progress if left unaddressed. Prioritizing ethical transparency, expanding public education, and incorporating a more diverse range of voices in the discussion could help drive broader acceptance and responsible use of BCI. Although this study uses advanced AI tools to offer valuable insights into public sentiment, certain limitations, such as potential biases and incomplete demographic data, should be acknowledged. Nonetheless, the findings serve as a valuable reference point for guiding future technological developments, informing policy making, and crafting effective communication strategies within the BCI sector.

## Supplementary material

10.2196/60859 Multimedia Appendix 1 Additional diagrams, tables, and figures.

## Abbreviations

AI: artificial intelligence
AR: augmented reality
BCI: brain-computer interface
DARPA: Defense Advanced Research Projects Agency
NLP: natural language processing
VADER: Valence Aware Dictionary and Sentiment Reasoner
VR: virtual reality
