# Supplementary material for: Public Perception of the Brain-Computer Interface Based on a Decade of Data on X: Mixed Methods Study
Source: JMIR Form Res. 2025 Jun 25;9:e60859. doi: 10.2196/60859 (PMC12242710; doi:10.2196/60859)
Supplement: Multimedia Appendix 1 [file formative-v9-e60859-s001.docx]

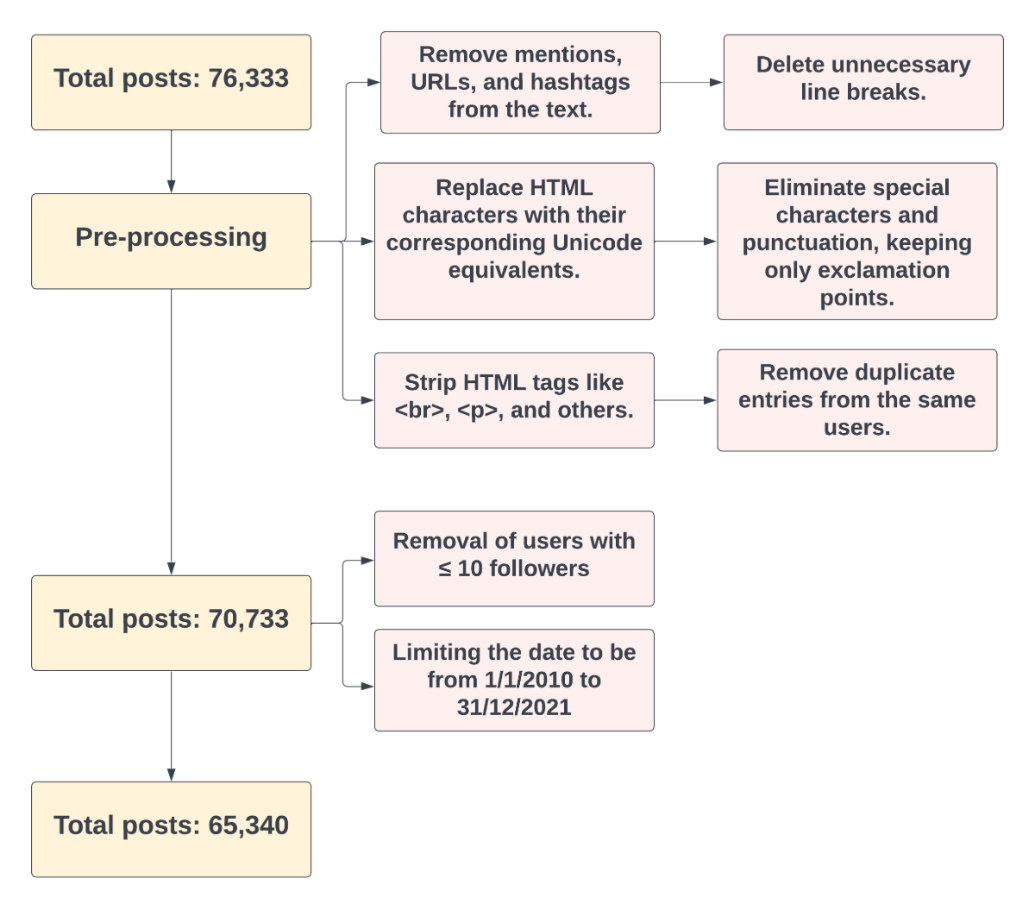


**Figure S1.** Data handling flowchart. Starting with a total of 76,333 posts. The pre-processing steps include removing mentions, URLs, hashtags, unnecessary line breaks, and duplicate entries, as well as stripping HTML tags and replacing special characters with Unicode equivalents. The process also eliminates posts from users with 10 or fewer followers and limits the dataset to posts between January 1, 2010, and December 31, 2021. After these steps, the final dataset is refined to 65,340 posts.

**Table S1.** Attributes used to classify users into biographic groups among those discussing BCI on X.

| Biographic group^a^ | Attributes |
| --- | --- |
| Scientific | “institution”, “scientist”, “academic”, “professor”, “student”, “researcher”, “university”, “research center” |
| Broadcasting | “journalist”, “journal”, “radio”, “bot”, “podcast”, “conference” |
| Entrepreneurship | “entrepreneur”, “sport”, “company”, “coach”, “CEO” |
| Clinical | “therapist”, “clinician”, “practitioner” |
| a: If the users' biography exhibits a cosine similarity score with the attributes of less than 0.05, the term "Others" is used. | |


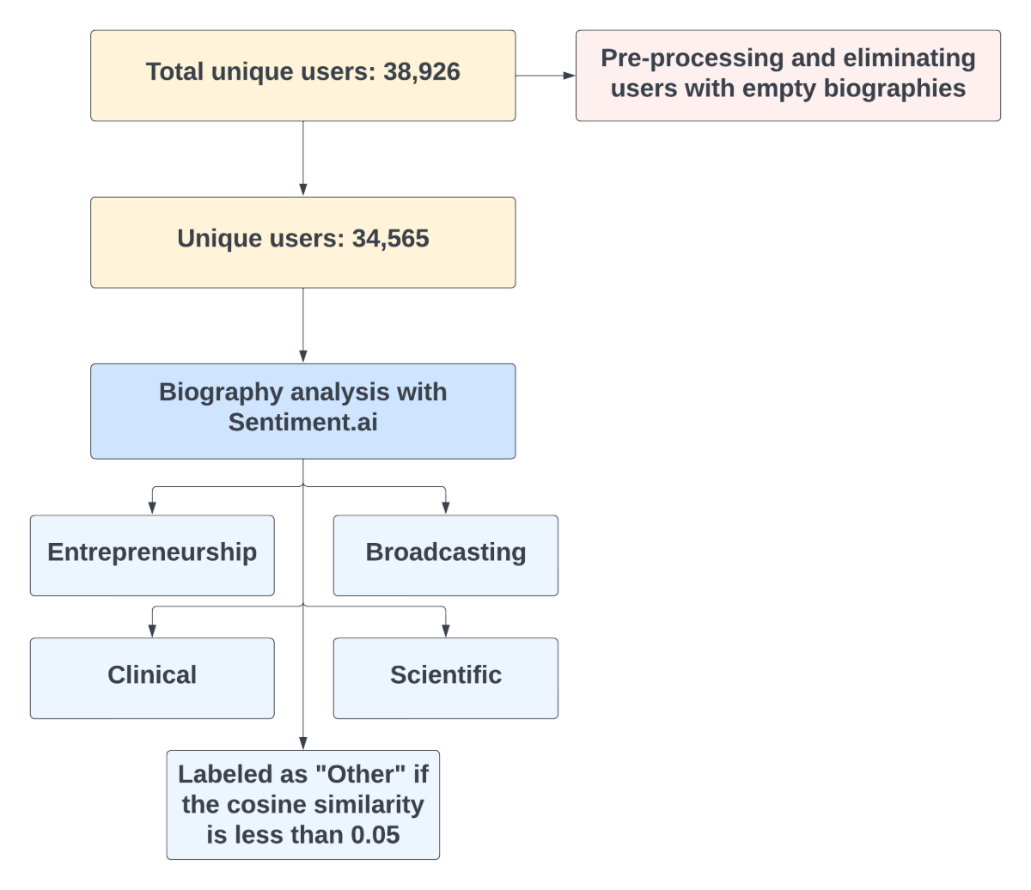


**Figure S2.** Biography analysis flowchart. Starting with a total of 38,926 unique users, pre-processing steps were applied to eliminate users with empty biographies, resulting in 34,565 users for analysis. These users’ biographies were analyzed using Sentiment.ai to classify them into four primary groups based on their attributes: Entrepreneurship, Broadcasting, Clinical, and Scientific. Users whose biographies did not meet a cosine similarity threshold of 0.05 with any group attributes were labeled as “Other.”


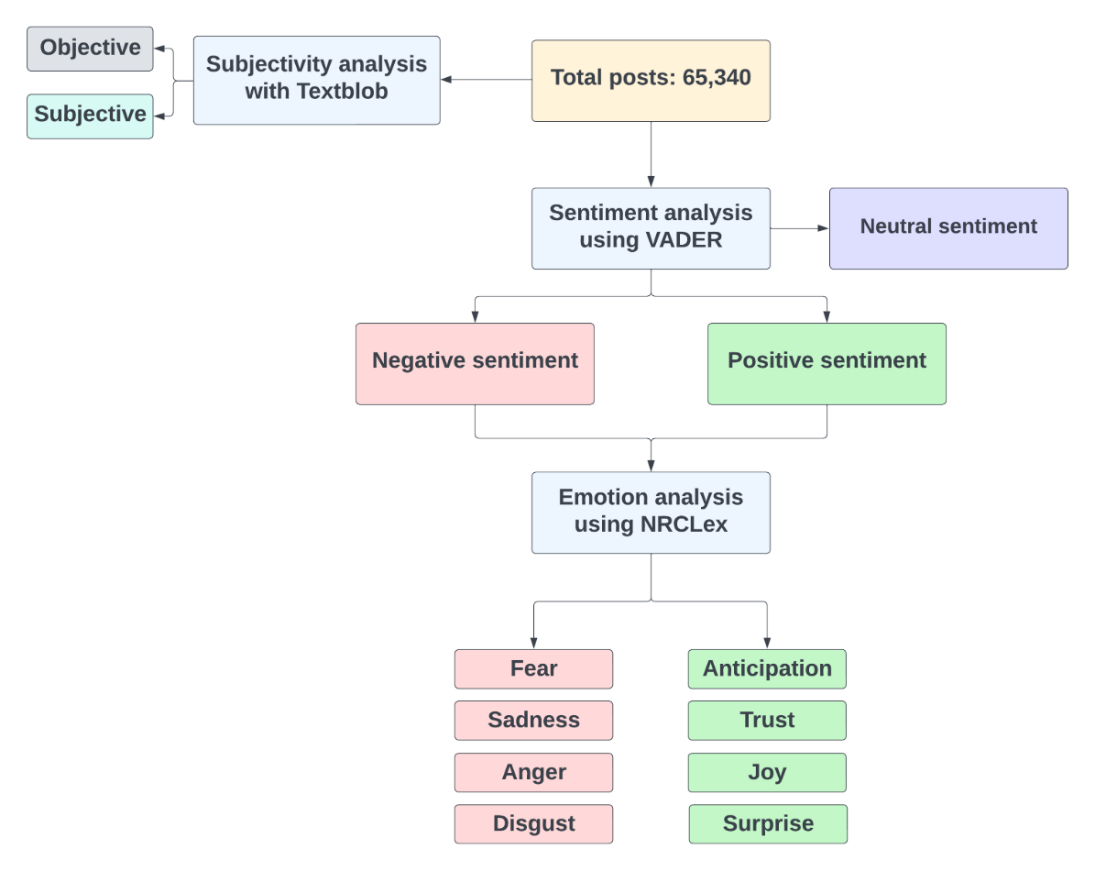


**Figure S3.** Sentiment & emotion analyses flowchart. A total of 65,340 posts discussing BCI on X were analyzed. The first step involved subjectivity analysis using TextBlob, categorizing the posts as either objective (subjectivity score < 0.5) or subjective (subjectivity score ≥ 0.5). Next, sentiment analysis using VADER was conducted to classify the posts into neutral sentiment (compound score < 0.05 and > -0.05), negative sentiment (compound score ≤ -0.05), or positive sentiment (compound score ≥ 0.05). Posts identified as having neutral sentiment were not analyzed further. Posts with negative or positive sentiment were subjected to emotion analysis using NRCLex. Negative emotions included fear, sadness, anger, and disgust, while positive emotions comprised anticipation, trust, joy, and surprise.


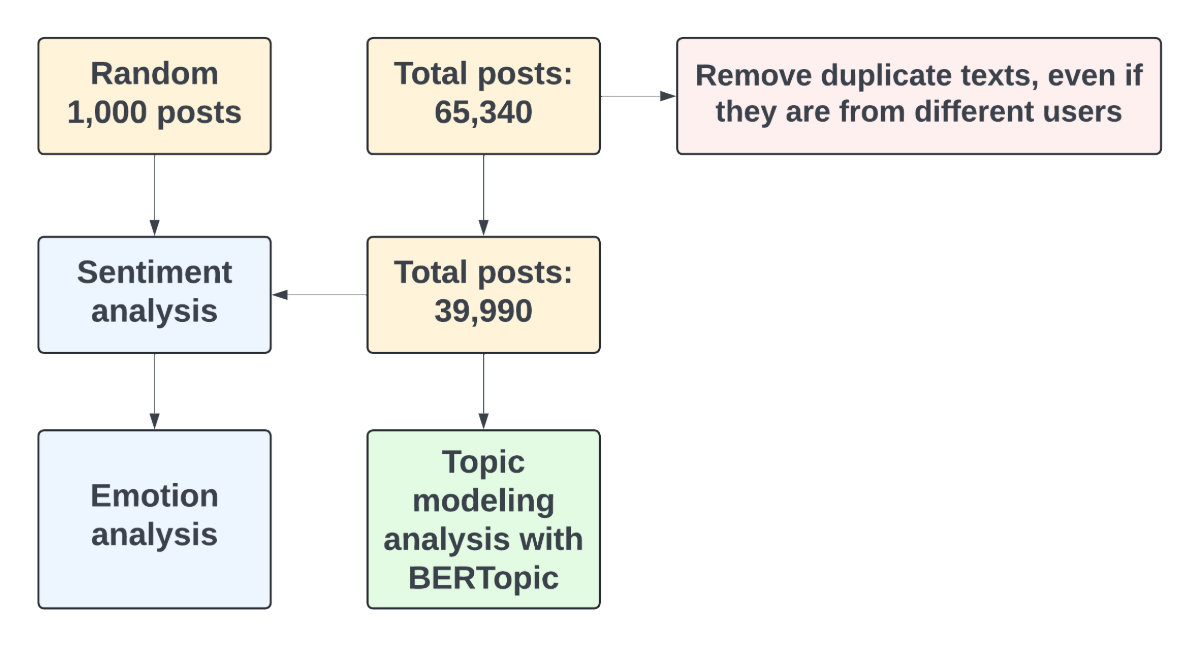


**Figure S4.** Topic modeling & sensitivity analyses flowchart. The analysis starts with 65,340 total posts discussing BCI on X. Duplicate texts are removed, even if they are from different users, resulting in 39,990 unique posts. This refined dataset then undergoes topic modeling analysis using **BERTopic.** A random subset of 1,000 posts is selected for validation purposes. This subset is subjected to sentiment analysis, followed by emotion analysis to ensure consistency and reliability of the findings from the overall dataset.


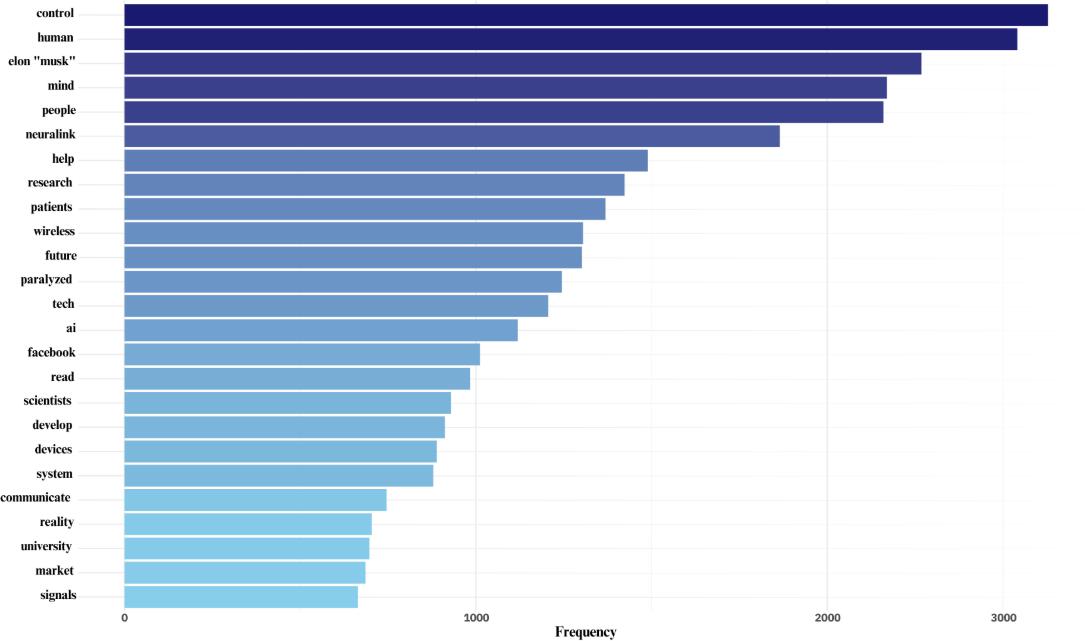


**Figure S5.** The frequency of 25 most used words in posts related to BCI discussions on X. The most frequently occurring word is “control,” followed by “human” and “elon ‘musk’.” Other highly frequent words include “mind,” “people,” and “neuralink,” indicating these topics are central in the posts. Additional words like “research,” “patients,” “future,” “tech,” and “ai” are also common, suggesting the inclusion of technological and scientific themes. The word “facebook” appears with moderate frequency, reflecting its mention in the context of BCI discussions.


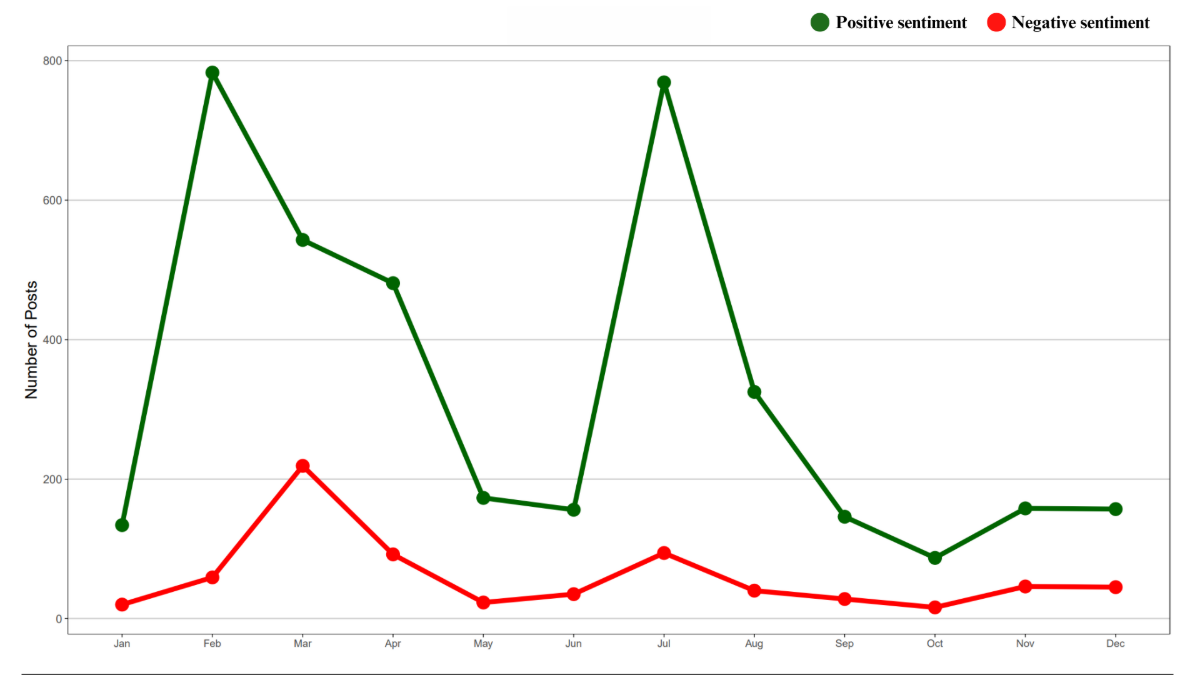


**Figure S6.** Monthly distribution of positive and negative sentiment posts discussing BCI on **X** throughout 2017. Positive sentiment (green line) posts peak significantly in February and July, while negative sentiment posts (red line) reach their highest point in March and maintain a relatively steady and lower presence through the year.


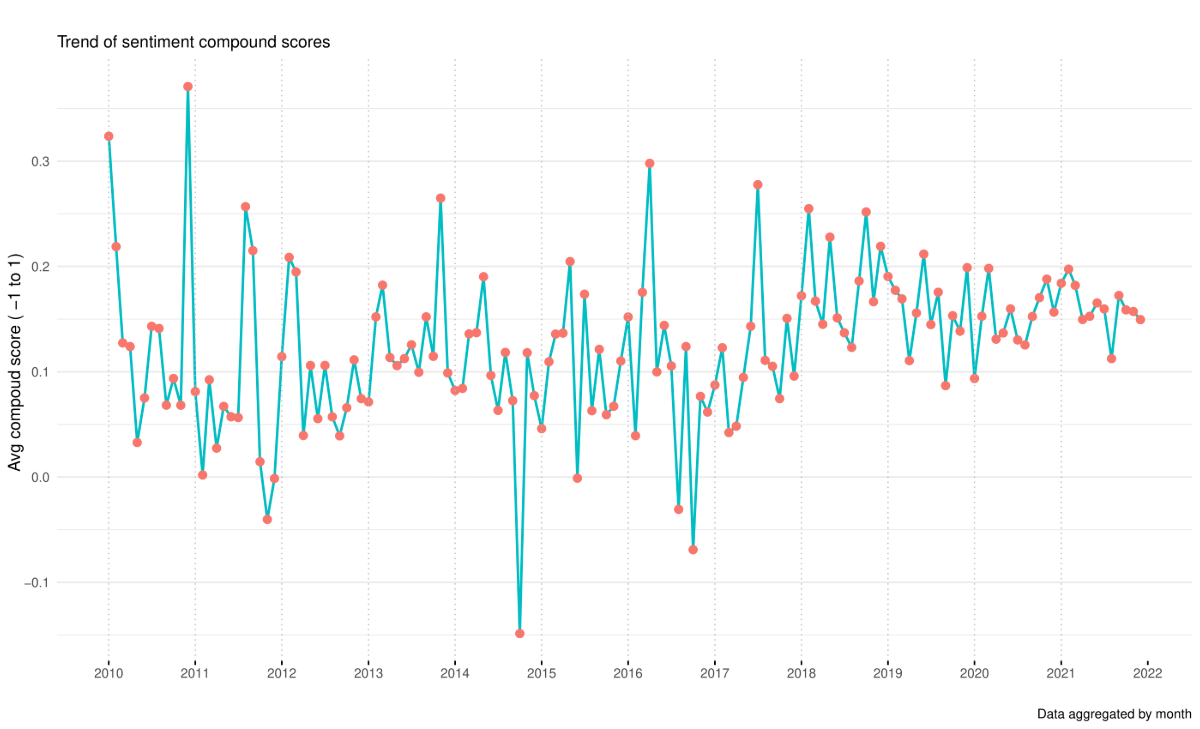


**Figure S7.** The trend of average sentiment compound scores over time, aggregated monthly related to BCI discussions on **X**. It shows a statistically significant positive trend throughout the study period, indicating a general increase in positive sentiment (Mann-Kendall Statistic = 0.266, tau = 0.266, P<.001).


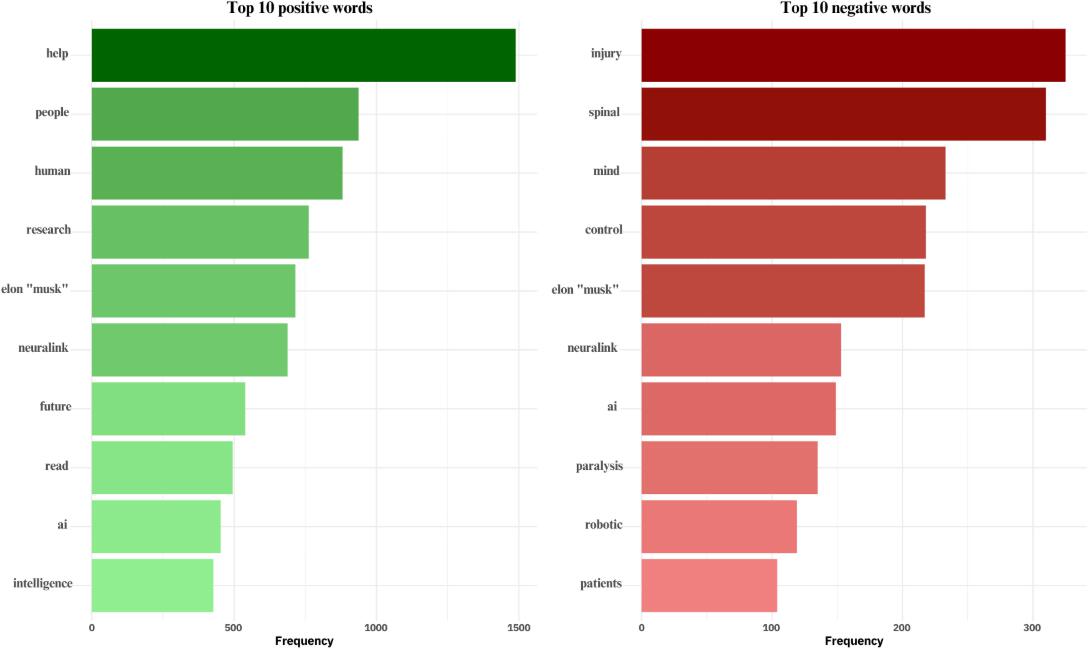


**Figure S8.** The top 10 most frequently used positive and negative words in posts discussing BCI on X. The left panel shows positive words, with “help” being the most frequent, followed by “people,” “human,” “research,” “elon ‘musk’,” “neuralink,” “future,” “read,” “ai,” and “intelligence.” The right panel displays negative words, with “injury” and “spinal” appearing most frequently, followed by “mind,” “control,” “elon ‘musk’,” “neuralink,” “ai,” “paralysis,” “robotic,” and “patients.”


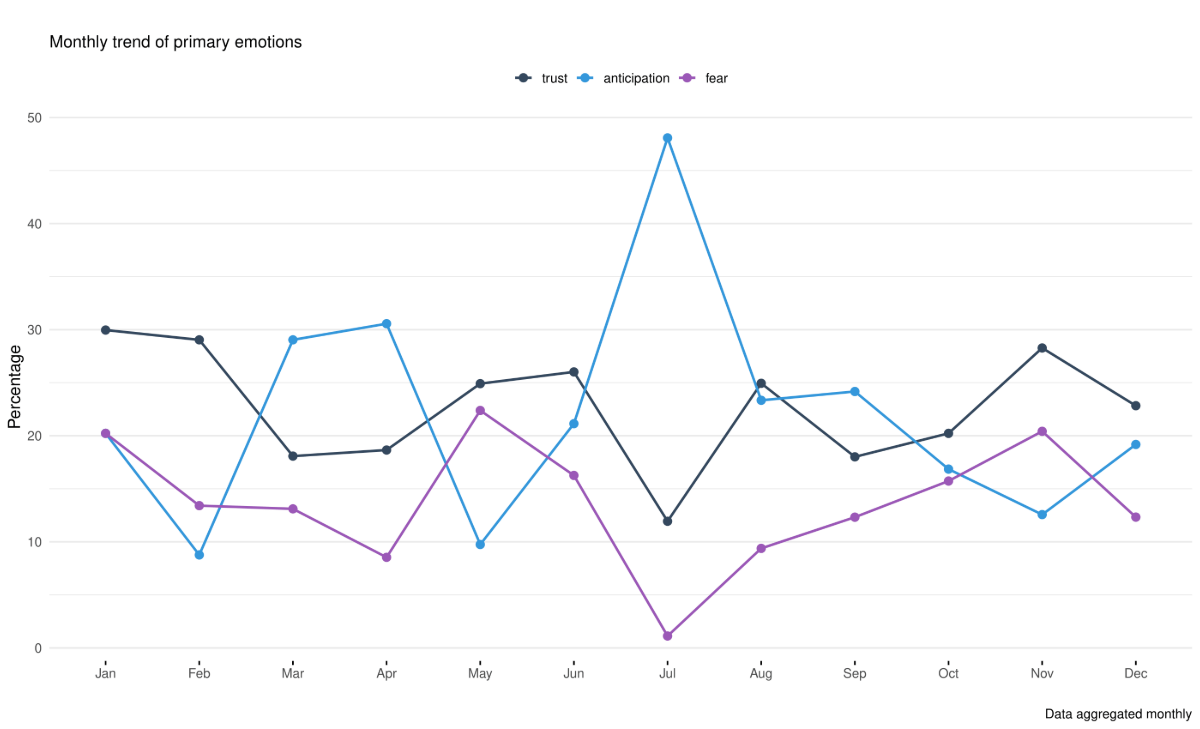


**Figure S9.** Monthly emotion percentages of anticipation (light blue), trust (dark blue), and fear (purple) related to BCI discussions on X for the year 2017. The emotion percentage is calculated as the frequency of each emotion divided by the total number of emotion occurrences for every month. Anticipation (light blue) demonstrates notable fluctuations, peaking significantly in July and showing a decline in February and May. Trust (dark blue) generally maintains a steady trend, with a peak in January and November, while showing a decline in July. Fear (purple) exhibits a lower, more stable pattern compared to the other emotions, with subtle rises and declines, peaking in May and dropping in July.


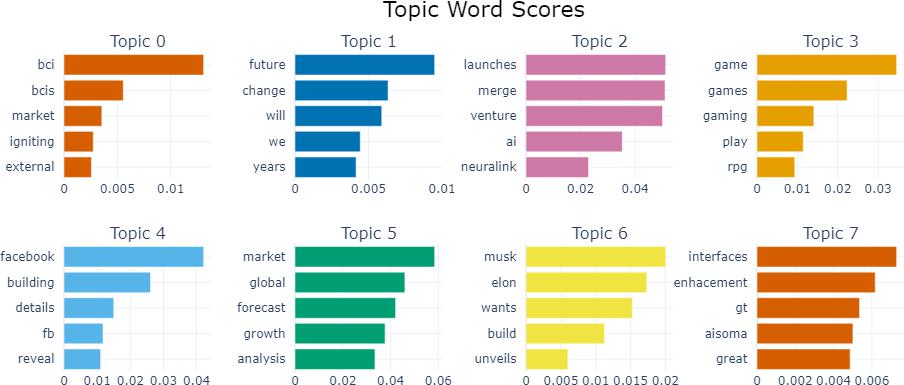


**Figure S10.** The word scores for the eight most relevant topics identified in BCI discussions on X. Each panel, labeled from Topic 0 to Topic 7, displays the most relevant words along with their scores. Topic 0 includes words such as “bci,” “bcis,” and “market.” Topic 1 features terms like “future,” “change,” and “years.” Topic 2 includes “launches,” “merge,” and “neuralink.” Topic 3 contains words such as “game,” “play,” and “RPG (role-playing game).” Topic 4 shows words like “facebook,” “building,” and “details.” Topic 5 includes terms such as “market,” “global,” and “growth.” Topic 6 features words like “musk,” “elon,” and “unveils.” Topic 7 includes “interfaces” and “enhancement.”

**Figure S11**. The word scores for the eight most relevant topics identified in BCI discussions with positive sentiment on X. Each panel, labeled from Topic 0 to Topic 7, displays the most relevant words and their scores. Topic 0 includes words such as “phd,” “research,” and “article.” Topic 1 features words like “my,” “fun,” and “want.” Topic 2 includes “bci,” “interested,” and “register.” Topic 3 contains words like “million,” “awards,” and “perfect.” Topic 4 shows words such as “VR” “AR,” and “reality.” Topic 5 includes “feel,” “again,” and “helps.” Topic 6 features words like “musk,” “elon,” and “neuralink.” Topic 7 includes “usual,” “commands,” and “everyday.”
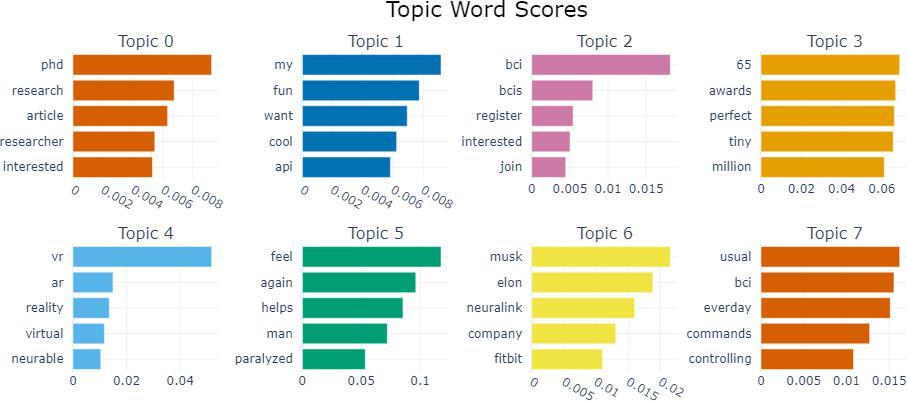


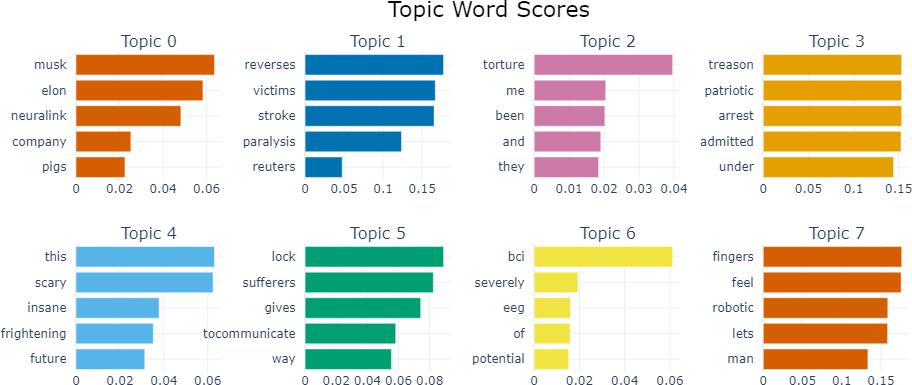


**Figure S12.** The word scores for the eight most relevant topics identified in BCI discussions with negative sentiment on X. Each panel, labeled from Topic 0 to Topic 7, displays the most relevant words and their scores. Topic 0 includes words such as “musk,” “elon,” and “neuralink.” Topic 1 features words like “reverses,” “victims,” and “stroke.” Topic 2 includes “torture,” “me,” and “been.” Topic 3 contains words like “treason,” “patriotic,” and “arrest.” Topic 4 shows words such as “this,” “scary,” and “insane.” Topic 5 includes “lock,” “sufferers,” and “gives.” Topic 6 features words like “bci,” “severely,” and “eeg.” Topic 7 includes “fingers,” “feel,” and “robotic.

**Table S2.** Analysis of Sentiment, Subjectivity, Primary Emotions, and Topics in BCI-Related Posts on ***X***.

| Post | Sentiment | Subjectivity | Primary emotions | Topic |
| --- | --- | --- | --- | --- |
| “Elon Musk launches Neuralink, a venture to merge the human brain with AI  Musk: "Over time I think we will probably see a closer merger of biological intelligence and digital intelligence." | Positive | Objective | Anticipation, Trust | Elon Musk’s involvement in BCI industry |
| “Brain-Computer Interface Can Help You Get In The Game: A simple visor can help you take home the gold” | Positive | Objective | Anticipation, Trust | Gamification of BCI |
| “Facebook is building a Brain-Computer interface for typing directly from your brain. Yes your brain. #F8 #Facebook” | Positive | Objective | Trust | Facebook’s involvement in BCI industry |
| “A new brain-computer interface for music composition, So cool! ALS patient technology expands into the music world.” | Positive | Subjective | Trust | Music production using BCI |
| “…the terrorist peoples are using voice transformers to communicate each other. Moreover, they have using brain computer interface method for terrorism.” | Negative | Objective | Anger, Disgust, Fear, Surprise | Malicious use of BCI technologies |
| “side-channel attack on a Brain Computer Interface (BCI): ...leak sensitive personal information such as passwords, PINs, whether a person is known to the subject, or even reveal emotions and thoughts.” | Negative | Subjective | Surprise | Hacking of BCI |
